# Thin-Film Composite Matrimid-Based Hollow Fiber Membranes for Oxygen/Nitrogen Separation by Gas Permeation

**DOI:** 10.3390/membranes13020218

**Published:** 2023-02-10

**Authors:** Daniel González-Revuelta, Marcos Fallanza, Alfredo Ortiz, Daniel Gorri

**Affiliations:** Departamento de Ingenierías Química y Biomolecular, Universidad de Cantabria, Av. de los Castros s/n, 39005 Santander, Spain

**Keywords:** gas separation, nitrogen, oxygen, hollow fiber membranes, dual-layer, selective membranes

## Abstract

In recent years, the need to reduce energy consumption worldwide to move towards sustainable development has led many of the conventional technologies used in the industry to evolve or to be replaced by new alternatives. Oxygen is a compound with diverse industrial and medical applications. For this reason, obtaining it from air is one of the most interesting separations, traditionally performed by cryogenic distillation and pressure swing adsorption, two techniques which are very energetically expensive. In this sense, the implementation of membranes in a hollow fiber configuration is presented as a much more efficient alternative to carry out this separation. The aim of this work is to develop cost-effective multilayer hollow fiber composite membranes made of Matrimid and polydimethylsiloxane (PDMS) for the separation of oxygen and nitrogen from air. PDMS is used as a cover layer but can also enhance the performance of the membrane. In order to compare these two materials, three different configurations are studied. First, integral asymmetric Matrimid hollow fiber membranes were produced using the spinning method. Secondly, by using dip-coating method, a PDMS dense selective layer was deposited on a self-made polyvinylidene fluoride (PVDF) hollow fiber support. Finally, the performance of a dual-layer hollow fiber membrane of Matrimid and PDMS was studied. Membrane morphology was characterized by SEM and separation performance of the membranes was evaluated by mixed-gas permeation experiments. The novelty presented in this work is the manufacture of hollow fiber membranes and the way Matrimid is treated. This makes it possible to develop much thinner dense layers than in the case of flat-sheet membranes, which leads to higher permeance values. This is a key factor when implementing this technology on an industrial scale. Membranes prepared in this work were compared to the current state of the art, reporting quite good performance for the dual-layer membrane, reaching O_2_ permeance of 30.8 GPU and O_2_/N_2_ selectivity of 4.7, with a thickness of about 5–10 μm (counting both selective layers). In addition, the effect of operating temperature on the membrane permeances has been studied experimentally; we analyze its influence on the selectivity of the separation process.

## 1. Introduction

In the past several decades, oxygen and nitrogen have become two compounds of high interest, especially at an industrial and medical level. Pure oxygen is commonly used in chemical and petrochemical processes, gas production, oil refining, power generation and in the glass industry. In a like manner, the applications of oxygen-enriched air include medical devices, combustion enhancement for furnaces, oxygen gas improvement in sewerage treatment plants, and undersea breathing [1,2]. Correspondingly, enriched and pure nitrogen also have various applications; inert gas in food processing, cryogenic agent, and as combustion diluent, to name some examples [3].

It is for this reason that the separation of oxygen and nitrogen from air has become one of the major topics to be investigated in the gas separation field. Until now, the techniques most widely used to perform this separation are cryogenic distillation and pressure swing adsorption (PSA). On the one hand, cryogenic distillation has the benefits of producing oxygen purity above 99% and a very high daily production, but it is only cost-effective for large-scale production due to the large amount of energy required to liquefy air by cooling and the high equipment cost covering heat exchangers, compressors, and refrigerants [4,5]. On the other hand, PSA can reach up to 95% oxygen purity, but it requires the use of pressure vessels and adsorbents, which makes it a cost-effective technology on a medium-to-large scale. In addition, it has a somewhat smaller production capacity than cryogenics because the need to use adsorbents limits its size capacity, mainly due to capital cost [6,7].

Another emerging method of oxygen/nitrogen separation that has gained importance in recent years is membrane separation. A membrane process to separate nitrogen from air inevitably produces oxygen-enriched air as a by-product. This method has the capability of producing a permeate gas containing 30–60% oxygen with a one-stage separation process [8], and it overcomes many of the barriers that cryogenics and pressure swing adsorption have; it has possibility of working on small scale, as well as lower energy and operational costs [6,7]. Despite these benefits, oxygen/nitrogen separation by membranes is not widespread on an industrial scale, and further development is still needed for it to compete with conventional techniques. Baker [9] listed the membrane separation types that were expected to be the highest priority 30 years ago. Separation of oxygen from nitrogen was among the top five priorities and had a wide margin for improvement. Even today, it is still expected to be one of the most important gas separations (and with the greatest economic investment) in the next two or three decades [10,11]. With this technology, high purities of oxygen and nitrogen can be achieved in several steps through the membrane module, or even through the possibility of forming a hybrid process in which a final cryogenic distillation step is added to achieve even higher purities.

There are two common kinds of membranes for this application: polymeric or ceramic. Ceramic membranes are capable of producing very high purity oxygen but exhibit low oxygen permeability and productivity and require high operating temperatures; this makes it difficult for them to become a real industrial option [12]. Separation with polymeric membranes is a more mature technology. That is why almost all membranes currently used in industrial applications are dense polymeric membranes, where mass transport is usually described by the solution-diffusion mechanism. This model is commonly used in separation processes involving the use of dense membranes and occurs in three steps: gas is transported through a nonporous polymer film or membrane by dissolving into the face of the membrane exposed to high gas pressure, diffusing through the polymer, and desorbing from the face of the membrane exposed to low pressure [10]. 

There are three major membrane module configurations uses in gas separation, namely, flat sheet, spiral wound, and hollow fiber. Traditionally, flat sheet and spiral wound configuration have been a good choice in order to obtain the transport parameters for different membrane materials, but they have certain drawbacks, such as poor gaseous flow pattern and low packing density. To handle this problem and to be able to implement the membranes at the industrial level, competing with cryogenics and pressure swing adsorption, it is necessary to maximize the surface-to-volume ratio, as well as working with high pressures [13,14]. Is for that reasons that important efforts are being made in this field for the development of new polymeric materials and their implementation in hollow fiber membrane modules [15,16,17].

Among the variety of families of polymers that have been studied as membrane materials are polycarbonates, cellulose acetate, polysulfone, polyethersulfone (PES), polyimides, polyetherimide, polypyrrolones, and silicone rubber. The characteristics that these polymers must fulfil to have a good performance are, above all, a high permeate flux and a high selectivity. In addition, they must also have high mechanical strength, high temperature resistance, chemical stability, and easy processability [18,19,20,21,22,23].

Typically, hollow fiber membranes for gas separation consist of a support and an active layer. This can act alone or can be coated with a layer of another material that provides protection but can also improve the performance of the whole membrane. These are called dual-layer membranes and have been widely studied in recent years [24,25,26]. Chong et al. [27] used two different coating materials, polydimethylsiloxane (PDMS) and poly(ether block amide) (PEBA), to improve the separation properties of polysulfone hollow fiber membranes. Membranes coated with PDMS exhibited an oxygen permeance of 18.3 GPU (gas permeation units) (1 GPU = 10^−6^ cm^3^(STP)/(cm^2^ s cmHg)) with a selectivity of 4.56, much higher than for PEBA, with values of 12.2 GPU and 3.11, respectively. On the other side, Chen et al. [28] attempted to form a thin cobalt chelating layer on top of polyurethane (PU) membranes. The O_2_/N_2_ selectivity was increased with the addition of this layer from 2.6 to 4.4, while oxygen permeance decreased drastically from 0.88 to 0.23 GPU.

Chen et al. [14] compared the performance of three hollow fiber membranes produced with different commercial polymers (polyethersulfone, polyetherimide, and polyimide). In all cases, the fibers were coated with PDMS to seal the defects on the outer surface. They also discussed the relationships between the gas separation performance of the hollow fibers and the intrinsic gas properties of the dense flat-sheet membranes. Their results showed, in terms of O_2_/N_2_ separation, that PES, polyimide (Matrimid), and polyetherimide (Ultem) have O_2_ permeances of 8.4, 3.9, and 2.9 GPU, while their selectivities were 5.3, 7.5, and 8.8, respectively. In the case of flat-sheet membrane configuration, they found lower permeance values due to thicker membrane thicknesses, while selectivity remained almost unchanged. 

As seen, polydimethylsiloxane (PDMS) and Matrimid are two polymers with very promising oxygen/nitrogen separation properties. In this way, this paper outlines the development of hollow fiber membranes for oxygen separation from air. To compare these two materials, three different membranes are studied. First of all, integral asymmetric Matrimid hollow fiber membranes were produced using the spinning method. Secondly, through the dip-coating method, a PDMS dense layer was deposited on a polyvinylidene fluoride (PVDF) hollow fiber support fabricated through the spinning method. Finally, the performance of a dual-layer hollow fiber membrane of Matrimid and PDMS was studied. The novelty presented in this work is the fabrication of hollow fiber membranes and the way of treating the polymers, specifically Matrimid. As mentioned, Matrimid usually presents high O_2_/N_2_ selectivity values, but relatively low permeances compared to other polymers, which makes its implementation at an industrial level a hindrance. This fact may be caused by the impossibility of developing very thin flat membranes (below 20 µm). Producing Matrimid hollow fiber membranes makes it possible to achieve a very thin dense active layer (less than 5 µm), which results in much higher permeances, as shown in the Section 3.

## 2. Materials and Methods

### 2.1. Materials

**Figure 1:**
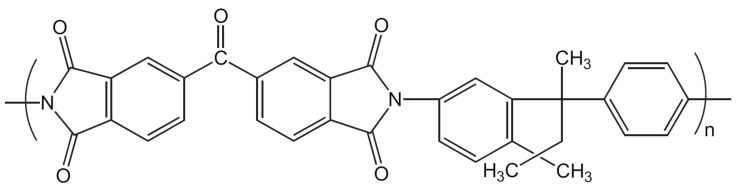
Chemical structure of Matrimid^®^ 5218 (adapted from ref. [29] with permission of Elsevier).

Matrimid^®^ 5218 (CAS nº 104983-64-4) was used to prepare the membranes, which was kindly provided by Huntsman (USA). The repeating chemical structure of Matrimid can be seen in Figure 1 [29]. Likewise, polyvinylidene fluoride (PVDF) (CAS nº 24937-79-9) was obtained from Arkema (Kynar^®^). 1-Methyl-2-pyrrolidone (NMP) (CAS nº 872-50-4) was used as solvent of Matrimid and PVDF and was provided by Sigma-Aldrich (Emplura^®^).

Polydimethylsiloxane (PDMS) (CAS nº 63148-62-9) and the crosslinking agent were supplied by Dow Corning (Sylgard^®^ 184). Hexane (CAS nº 110-54-3) was used as solvent of PDMS and it was obtained from Honeywell (purity ≥ 99%). Polyvinylpyrrolidone (PVP) (CAS nº 9003-39-8) was supplied by Sigma-Aldrich.

The gases used, namely, oxygen (purity > 99.995%), nitrogen (purity ≥ 99.999%), and helium (purity ≥ 99.999%), have been provided by Nippon Gases España, S.L.U.

### 2.2. Spinning

**Figure 2:**
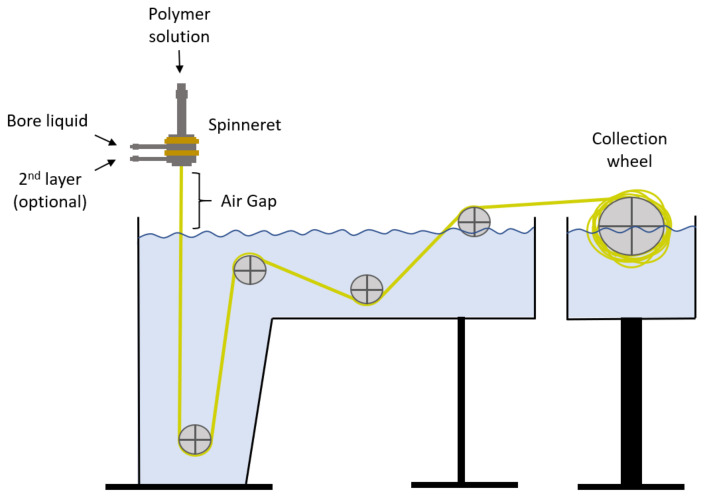
Schematic diagram of spinning process.

In this work, the dry/wet spinning method is used for the manufacture of hollow fiber membranes. This system consists in the extrusion of the polymer with the help of a spinneret, which first contacts the air (air gap) and then enters the coagulation bath. This method is quite simple and highly reproducible, which makes it ideal for this type of application. Two types of fibers have been made, one of Matrimid and the other of PVDF. The latter is the support on which the PDMS layer is placed. The coagulation tank and the entire pulley system is homemade, and the spinneret was supplied by EMI Twente (Enschede, The Netherlands). A schematic diagram of the spinning method is shown in Figure 2.

On the one hand, the dope solution employed for the Matrimid hollow fiber fabrication was composed of 20 wt.% Matrimid and 80 wt.% NMP. Matrimid flakes were slowly added into NMP under mechanical stirring at 60 °C for 6 h. Subsequently, when completely dissolved, it was degassed for 24 h at room temperature. When the solution was ready to be extruded, both the polymer solution and the bore liquid were placed in stainless steel syringes and pumped into the spinneret. The composition of the bore liquid used was 20 wt.% NMP and 80 wt.% Milli-Q water, in order to allow a sufficiently low demixing speed and extend the contact between NMP and the polymer during coagulation, resulting in a high pore density and large macrovoids [30,31,32]. The ultimate goal in Matrimid hollow fiber membranes is to achieve a porous structure with a thin dense selective outer layer, which is why water at room temperature is used as the external coagulant. Once extruded, the hollow fibers are left in water for at least 72 h to finish coagulation. Subsequently, to dry them for use, they are left in a methanol bath for 20 min and finally subjected to vacuum for at least 6 h.

In addition, PVDF solution was prepared with 16 wt.% PVDF, 1 wt.% PVP, and 83 wt.% NMP. PVP is added as an additive because it increases the precipitation rate due to its hydrophilic nature. This may result in a formation of larger macrovoids, achieving higher porosity in the final membranes [33]. PVDF pellets were slowly added to the NMP under stirring at 70 °C, and finally, PVP was added and left stirring at this temperature for 8 h. Lastly, the solution was degassed for 24 h at room temperature. In this case, a mixture containing 20 wt.% ethanol and 80 wt.% water was used as bore liquid with the same objective as in the case of Matrimid, i.e., to achieve a high pore density and macrovoids in the inner side of the hollow fiber membrane. This PVDF hollow fiber membrane was used as support for another material selective layer (i.e., PDMS), so it is desirable to exert as little resistance as possible in compounds permeation. Therefore, the outer layer must also be highly porous, and to achieve this, water was used as coagulation bath at 30 ºC. It is desirable to work with a high temperature coagulation bath to obtain finger-like pores, but without reaching a temperature that compromises the mechanical resistance of the hollow fiber membrane [34]. Similarly, the hollow fibers are left in water for at least 72 h with frequent replacement of fresh water, in order to completely wash away the solvent. Subsequently, they are left in an ethanol bath for 20 min in order to displace the water and to facilitate the drying process. Finally, they are subjected to vacuum. Further parameters of the spinning process for each membrane can be found in Table 1.

### 2.3. Dip-Coating

Dip-coating is carried out after spinning and when hollow fiber membranes have completely dried. This method consists of impregnating a membrane with another material to create a layer on top of it. To do this, the hollow fiber membranes are immersed for a few seconds in the polymer solution and removed, thus creating a very thin layer on the outer surface of the hollow fiber. Normally, in the hollow fiber spinning process, microdefects and imperfections can occur at some points of the membrane, and this may cause a considerable loss in selectivity [35]. So, this process can be carried out to minimize surface defects but also to provide the membranes with a selective dense layer, improving separation performance [27,36]. 

**Figure 3:**
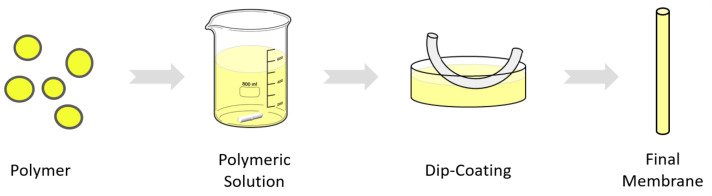
Dip-coating process scheme.

In this work, the dip-coating method is used, on the one hand, to prepare a thin PDMS membrane on the PVDF support, and on the other hand, to prepare a dual-layer membrane of Matrimid and PDMS. The latter is a membrane consisting of two layers of different materials, one of them added by dip-coating. In both cases, the coating procedure is carried out in the same way. A mixture of PDMS is prepared with the curing agent in a ratio of 1:10, and then, by adding the corresponding amount, a solution with a concentration of 10% is prepared using hexane as solvent and stirred with a magnetic stirrer at room temperature for 1 h. The viscosity of the solution is then measured using a rotational viscometer. It is important to know the viscosity because it is the key factor that will ultimately determine the thickness of the coating layer on the hollow fiber. Previous studies have also found this relationship of the concentration/viscosity with the final thickness of the membrane [37,38]. The solution is poured onto a 30 cm long glass container and dip-coating is performed. The length of the fibers for dip coating is about 25 cm, enough to produce a module of 15 cm in length, and they are dipped into the polymer solution for 5 s, 3 times. Finally, the hollow fibers are left to dry in a vertical position in oven at 100 ºC for 1 h so that the polymer cures (Figure 3). 

### 2.4. Membrane Characterization

The membranes have been morphologically characterized by scanning electron microscopy (SEM). These measurements have been carried out in the laboratory of the Materials Science and Engineering Division (LADICIM), at the University of Cantabria, with a Zeiss EVO MA15 microscope. To perform a precise cut of the membranes, they were frozen with liquid nitrogen, obtaining a clean fracture. In this way, the cross section, as well as the external and internal surface, could be properly observed.

### 2.5. Gas Permeation Experiments

**Figure 4:**
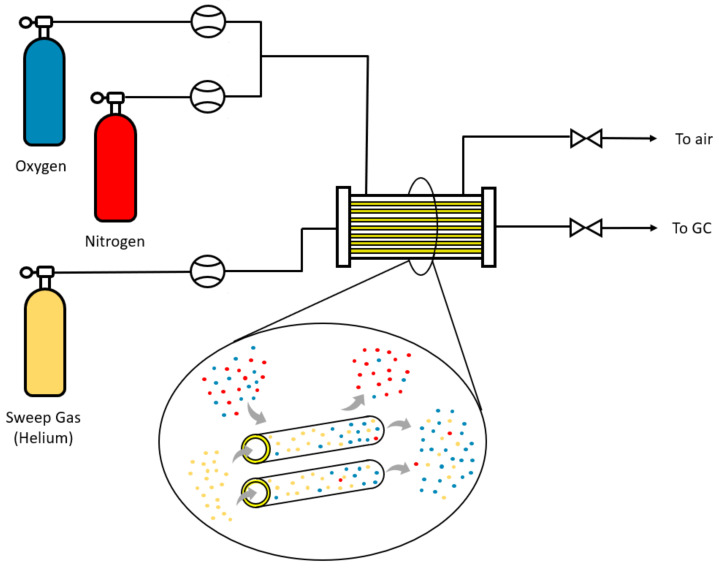
Scheme of the gas permeation process.

The performance of the membranes in gas permeation was tested in an experimental setup at lab scale. Modules were constructed with a shell of stainless steel and 5 fibers with a length of 15 cm were inserted into it; the ends were sealed with epoxy resin to avoid possible leaks. The scheme of the gas permeation process is shown in Figure 4. A synthetic mixture of oxygen and nitrogen with a 50:50 mol ratio is fed through the shell side, where a certain pressure is applied to produce a pressure gradient between the two sides of the membrane. The permeate is collected on the lumen side of the fiber, and helium is used as sweep-gas in order to continuously remove the permeating oxygen and nitrogen and to always have a maximum driving force (partial pressure gradient). In addition, experiments have also been carried out in which pure oxygen and nitrogen were used as feed. 

The final objective is to obtain the permeance of each membrane, which is the pressure-normalized flux (i.e., transport flux per unit transmembrane driving force), and to calculate it. The flux of each component through the membrane must first be obtained as:

(1) $$J_{i}=\frac{Q_{i}}{D_{LM}\;\pi \;L\;N}\;$$

where $J_{i}$ is the permeation flux across the membrane of each compound, $Q_{i}$ is the flow of each compound permeating across the membrane, $D_{LM}$ is the logarithmic mean diameter of the hollow fibers, L is the length of the hollow fibers, and N is the number of fibers placed in the module. Once the flux of each compound is obtained, the permeance of the membrane to each compound can be deduced as follows:

(2) $$P_{i}=\frac{J_{i}}{\Delta p_{i}}\;$$

where $P_{i}$ is the permeance of the membrane, and $\Delta p_{i}$ is the partial pressure gradient across the membrane. For a hollow fiber membrane module working in parallel flow mode (concurrent), the driving force is given by:

(3) $$\Delta p_{i}=\frac{\left(p_{i,feed}-p_{i,permeate\left(0\right)})-(p_{i,retentate}-p_{i,permeate}\right)}{LN\left(\frac{p_{i,feed}-p_{i,permeate\left(0\right)}}{p_{i,retentate}-p_{i,permeate}}\right)}$$

where $p_{i,feed}$ is the partial pressure of component i in the feed, $p_{i,permeate\left(0\right)}$ is the partial pressure of component i in the permeate at an initial position, $p_{i,retentate}$ is the partial pressure of component i in the retentate, and $p_{i,permeate}$ is the partial pressure of component i in the permeate. Finally, selectivity ($\alpha_{i/j}$) is defined as the ratio between the gas pair permeances of components through the membrane:

(4) $$\alpha_{i/j}=\frac{P_{i}}{P_{j}}\;.$$

The experiments were carried out in a temperature range between 20 and 80 °C and at partial pressures gradients between 0.5 and 2.5 bar. Table 2 shows feed and sweep gas flows used in this work, as well as total feed and permeate pressures. A chromatograph model HP-Agilent 6890 equipped with a TCD detector was used to measure the concentrations of each component in the permeate and retentate streams. In order to minimize the experimental error, the measurement of each pressure and temperature was repeated 5 times. In addition, on each membrane module tested, another module with the same membrane characteristics was manufactured to confirm its reproducibility.

## 3. Results and Discussion

### 3.1. Morphology Study of Hollow Fiber Membranes

#### 3.1.1. Morphology Study of Matrimid Hollow Fiber Membrane

**Figure 5:**
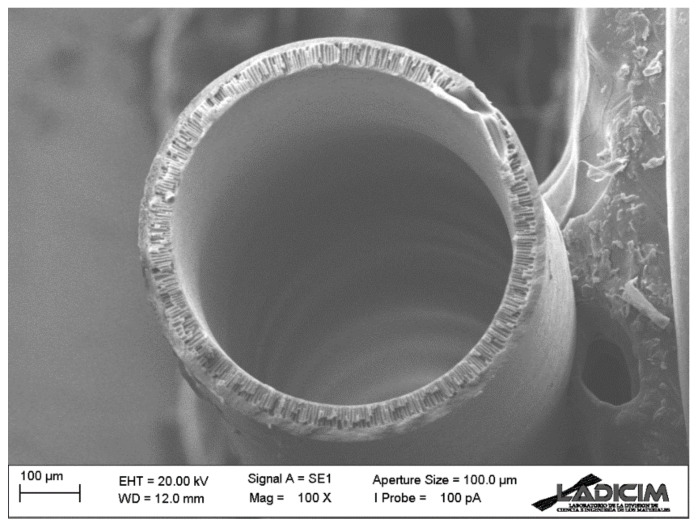
SEM image of the cross-section of the Matrimid hollow fiber membrane.

**Figure 6:**
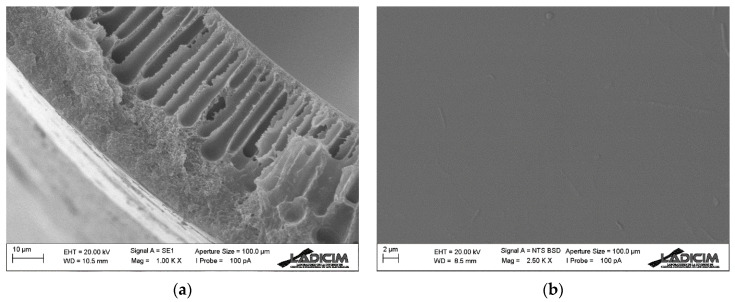
SEM images of Matrimid hollow fiber membrane: (**a**) cross-section detail and (**b**) external surface.

The morphology of the spun Matrimid hollow fiber membrane was examined by SEM. Figure 5 and Figure 6 show details of the membrane morphology. The hollow fiber has an external diameter of about 680 μm, while the wall thickness is 50 μm. As can be seen, finger-like pores dispersed in a regular way have formed along the membrane section, but there are also sponge-like pore areas in the central and external parts. Such porosity is evenly and symmetrically distributed along the entire section, giving the hollow fiber membrane adequate mechanical strength for the applications to be used while, at the same time, exerting almost no resistance for the gas transport process. Porosity in the inner layer has been achieved thanks to the bore liquid composition, as well as its flow rate in the spinning process. The mixture of NMP (20 wt.%) and milli-Q water in the bore liquid allows a slower precipitation and results in such porosity. Figure 5 shows the SEM image of the complete cross-section of the Matrimid hollow fiber membrane.

On the outer side, tap water is used in order to achieve rapid precipitation, as opposed to the inner layer. This results in the formation of a dense layer on the outside of the fiber, which is the one that actually carries out the separation of the compounds [39,40]. With a close look at the outer dense selective layer in Figure 6a, it is very difficult to distinguish, and therefore to define exactly the thickness of this layer, but its size is minimal (less than 5 µm). As shown in Figure 6b, the outer layer is completely smooth, without pores. Since it is such a thin layer, microdefects may have formed during the spinning process, in which case they are not visible in the SEM images. 

#### 3.1.2. Morphology Study of PVDF/PDMS Hollow Fiber Membrane

In order to obtain the performance of a dense PDMS membrane in gas separation, a PVDF support is used. This support must be as porous as possible in order not to have any effect on the separation, and to ensure that all the resistance to mass transfer is due to the PDMS layer, which is coating the support on the external side of the fiber. 

**Figure 7:**
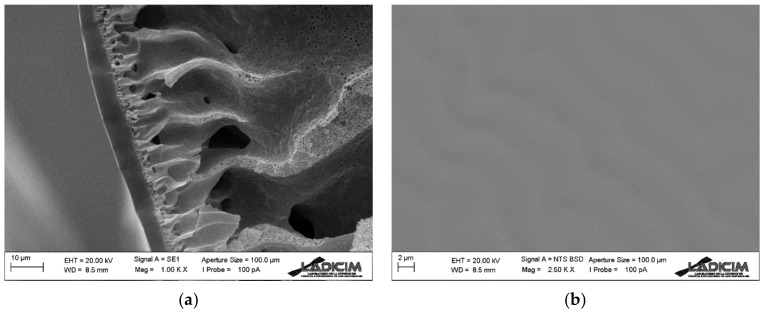
SEM image of PVDF-PDMS hollow fiber membrane: (**a**) cross section detail and (**b**) external surface.

Figure 7 shows the cross section and the outer surface of the PVDF hollow fiber support together with an ultrathin PDMS outer layer. On the one hand, it has been possible to obtain a PVDF hollow fiber with high mechanical strength, but at the same time with high porosity and large macrovoids along its cross section. This has been achieved by the addition of a small amount of PVP to the spinning dope, which favors a rapid precipitation. Moreover, due to the conditions used in its spinning—for example a minimum air gap—it does not have any dense outer layer, which is crucial for our process’s purpose. The use of a very small air gap means that the viscosity of the extruded solution does not increase considerably until it enters the coagulation bath, which allows better finger-like pores to form through the membrane and thus no dense outer layer to form [40]. The PVDF support has an external diameter of about 760 µm and a wall thickness of 170 µm. 

On the other hand, it can be observed how a PDMS dense thin layer has been deposited on the outside of the hollow fiber membrane. The thickness of this layer is around 3 µm.

#### 3.1.3. Morphology Study of Dual-Layer Hollow Fiber Membrane (Matrimid/PDMS)

**Figure 8:**
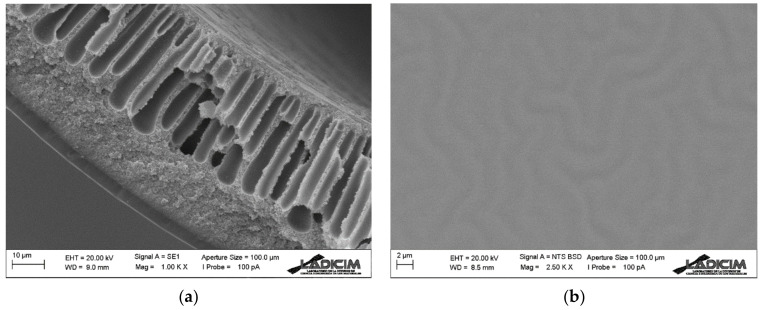
SEM analysis of dual-layer Matrimid/PDMS hollow fiber membrane: (**a**) cross-section detail and (**b**) external surface.

The morphology of the Matrimid hollow fiber membrane coated with an external PDMS layer can be seen in Figure 8. The cross section of the Matrimid membrane is the same as the one described in Section 3.1.1 of this work since the same one has been used, and the PDMS layer that covers it has been deposited by dip-coating and has similar characteristics to the one described in Section 3.1.2. As a result, a composite hollow fiber membrane is obtained with a Matrimid support, having a very thin dense layer of Matrimid and a very thin last PDMS coating. In other words, the end result is a Matrimid and PDMS dual-layer membrane.

Since the Matrimid hollow fiber membrane may have micro-defects, the PDMS coating serves the function of both covering these defects and providing a second dense separative layer to the membrane.

### 3.2. Separation Performance of Membranes

The performances of the three different membranes developed in this work were evaluated in terms of permeance and selectivity. For this, it is necessary to obtain the ratio of the flux of each component through the membrane and the partial pressure gradient of both of them across the membrane. Since it is very difficult to define exactly the dense layer thickness of the membranes developed, permeance has been chosen as the parameter with which to evaluate their performance. Permeance is a parameter that can be evaluated from experimental data without knowing the membrane thickness, unlike permeability, whose calculation requires knowledge of the membrane thickness. Membranes have been tested at a temperature range between 20 and 80 °C because this is the range in which these polymers perform optimally. Above that temperature, the polymers may lose their separation properties or even burn.

#### 3.2.1. Performance of Matrimid Hollow Fiber Membrane

**Figure 9:**
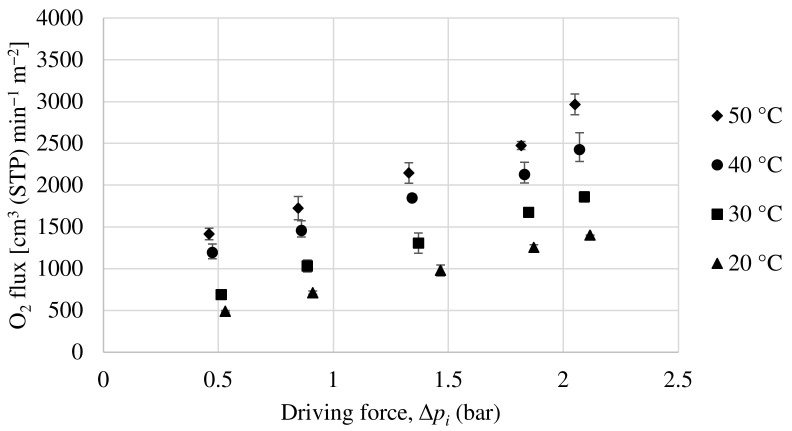
Evolution of oxygen flux as a function of partial pressure gradient for Matrimid hollow fiber membrane.

**Figure 10:**
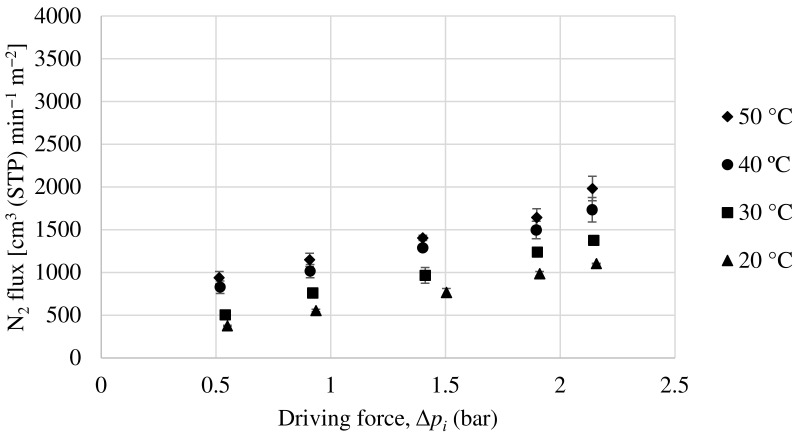
Evolution of nitrogen flux as a function of partial pressure gradient for Matrimid hollow fiber membrane.

In order to evaluate the permeability of the Matrimid hollow fiber membrane, experiments have been carried out with a pure oxygen and nitrogen feed and with a 50:50 mol ratio mixture feed of both compounds. Figure 9 and Figure 10 show the oxygen and nitrogen flux, respectively, versus the partial pressure gradient between the feed and the permeate for a temperature range from 20 to 50 °C and for the mixture feed. As can be seen, as the pressure gradient increases, both oxygen and nitrogen fluxes through the membrane increase monotonically, with the oxygen flux being notably higher, demonstrating that this is an oxygen-selective membrane. According to equation 2, the ratio between the partial flux and the driving force (making the relevant unit changes) will result in the membrane permeance (GPU). In relation to this, the permeance increases with increasing temperature for both oxygen and nitrogen. This is because a higher temperature improves the mobility of the polymeric chains, resulting in a faster gas diffusion through the membrane.

In view of the results, Table 3 shows a comparison of the evolution of oxygen permeance, nitrogen permeance, and selectivity of the Matrimid hollow fiber membrane for both feeding cases, pure gases, and mixture. It can be seen that the permeance for both components increases considerably with temperature, while the selectivity towards oxygen is only slightly increased. The advantage of this behavior for the practical application of these membranes is that the permeance can be increased by simply increasing the working temperature without compromising the selectivity. These permeance values for both compounds have been obtained for partial pressure ranges from 0 to 2.5 bar. As can be seen, the permeances and selectivity values obtained are quite similar for both types of feed.

Matrimid is a material that has been studied extensively in the literature, mostly in flat sheet configuration. Guiver et al. [41] characterized the performance of Matrimid flat sheet membranes, obtaining an oxygen permeance of 0.02 GPU and an O_2_/N_2_ selectivity of 7. Later, Xiao et al. [42] performed gas separation tests, including oxygen and nitrogen, with Matrimid and brominated Matrimid flat sheet membranes. As a result, they obtained an oxygen permeance of Matrimid of 0.035 GPU and an O_2_/N_2_ selectivity of 6.8.

The way a material is processed when synthesizing membranes is crucial in the final separation performance. Thus, when developing Matrimid membranes in hollow fiber configuration as has been conducted in this work, the results are far from those reported in the literature for flat sheet membranes. There are other authors who have worked with Matrimid membranes in hollow fiber configuration. Favvas et al. [43] developed Matrimid hollow fiber membranes as precursors for later carbonization. For the former ones, they found an oxygen permeance of 34.4 GPU and a selectivity of 1.5. Moreover, Ding et al. [44] fabricated hollow fiber membranes of polysulfone and Matrimid, obtaining an oxygen permeance of 15.2 GPU, with an O_2_/N_2_ selectivity of 2.09. The reason why lower selectivity and higher permeability are obtained working with hollow fiber membranes may be due to the fact that the selective dense layer is so thin that small pinholes can be produced along it, which may lead to these results.

#### 3.2.2. Performance of PVDF/PDMS Hollow Fiber Membrane

In order to characterize the performance of the PDMS used, a hollow fiber membrane of this material was fabricated on a porous PVDF support. The thickness of this active layer of PDMS was about 70 µm. In the same way, its oxygen and nitrogen permeance and its O_2_/N_2_ selectivity were obtained. The self-made PVDF support has been used and has been found to be completely porous, so it exerts a minimum resistance to the mass transfer of components through it. The objective of this choice is not to influence the performance of the separation process and to obtain results in which the PDMS dense layer is the only one that performs the separation. 

This membrane has been tested in a temperature range between 30 and 50 °C and with a 50:50 mol ratio mixture feed of oxygen and nitrogen. Table 4 summarizes the O_2_ and N_2_ permeances and O_2_/N_2_ selectivity obtained. As can be seen, the selectivity is a factor that is not influenced by temperature. This result agrees with that of Pian et al. [45], who developed ceramic hollow fiber-supported PDMS membranes and studied their oxygen and nitrogen permeance, looking at the influence of temperature, membrane thickness, and pressure difference. They found that the permeance increased by only 20% when the temperature was increased from 20 to 70 °C, while the O_2_/N_2_ selectivity remained almost constant. They obtained an O_2_/N_2_ selectivity for PDMS of 2, which is in agreement with the results obtained in this work, although it is somewhat lower. In another work, Sadrzadeh et al. [46] studied the sorption, diffusion, and permeation properties of PDMS, and found an ideal O_2_/N_2_ selectivity of 1.8.

#### 3.2.3. Performance of Dual-Layer Hollow Fiber Membrane (Matrimid/PDMS)

**Figure 11:**
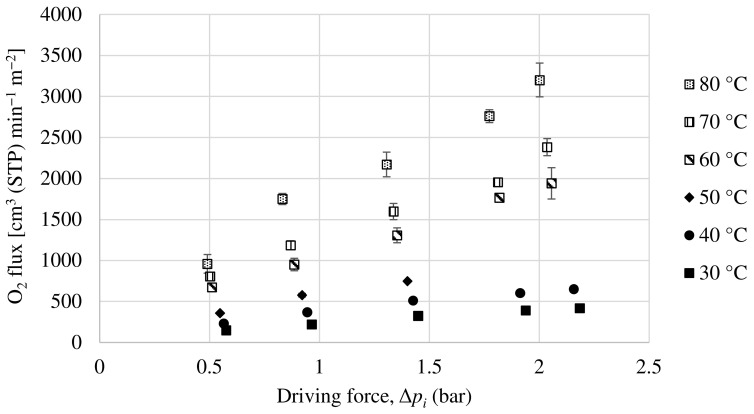
Evolution of oxygen flux as a function of partial pressure gradient for dual-layer Matrimid/PDMS hollow fiber membrane.

**Figure 12:**
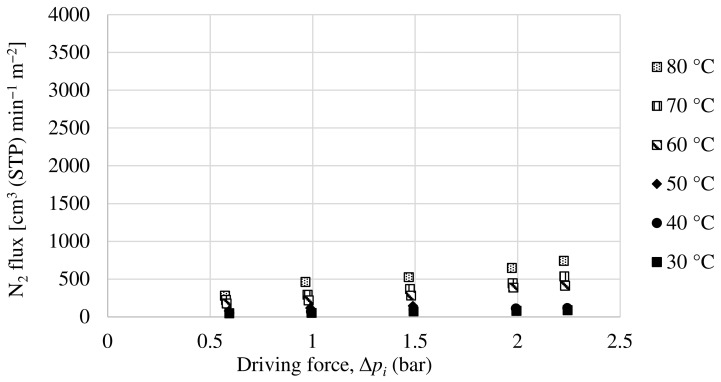
Evolution of nitrogen flux as a function of partial pressure gradient for dual-layer Matrimid/PDMS hollow fiber membrane.

A Matrimid hollow fiber dual-layer membrane with a thin PDMS coating has been developed. The Matrimid membrane used as precursor is the same as the one in Section 3.2.1, with the addition of the PDMS layer in order to completely seal the possible pinholes that the dense Matrimid layer may have. For this membrane, experiments have been carried out with a pure oxygen and nitrogen feed and with a 50:50 mol ratio mixture feed of both compounds. The partial fluxes for oxygen and nitrogen as a function of the driving force for the mixture feed are shown in Figure 11 and Figure 12. In the same way as the others, the oxygen and nitrogen permeance of this membrane has been obtained experimentally, as well as the selectivity at different temperatures. 

Table 5 summarizes the permeance and selectivity obtained with this membrane for the two feeding types studied, pure gases and mixture, the results for both types being quite similar. In this case, the permeance increases with increasing temperature, while the selectivity decreases slightly. As can be seen, the oxygen selectivity values obtained are significantly higher than those previously obtained with the same Matrimid membrane without PDMS coating, while the oxygen permeance values are less affected (although they decrease somewhat). These O_2_/N_2_ selectivity values are much closer to those previously mentioned for the Matrimid, which leads to the conclusion that the Matrimid hollow fiber membrane had pinholes in its dense layer and that by adding the PDMS coating, they have been sealed, obtaining a membrane with a good performance in oxygen/nitrogen separation. Thus, our results are in agreement with several studies that have shown the usefulness of applying a thin coating of a rubbery polymer such as PDMS as a protective layer that at the same time seals possible defects, improving selectivity at the cost of a certain decrease in permeances [47,48,49]. 

### 3.3. Temperature Dependence 

**Figure 13:**
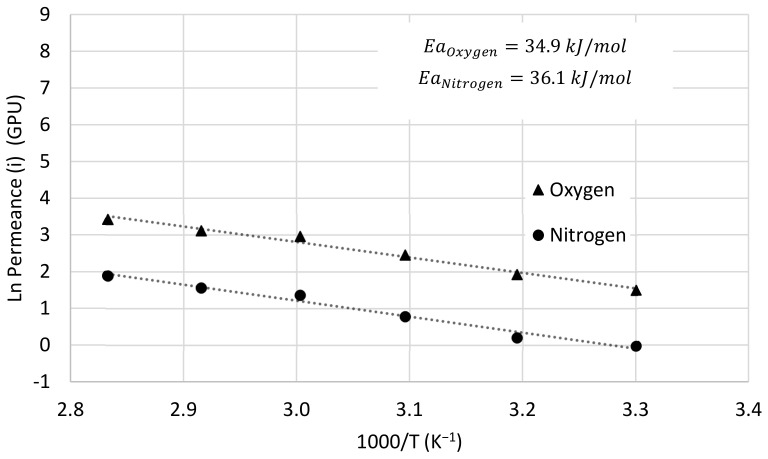
Arrhenius plot for oxygen and nitrogen permeance of dual-layer Matrimid/PDMS hollow fiber membrane when oxygen and nitrogen mixture is fed.

Attending to the effect of temperature on O_2_ and N_2_ permeance, it is possible to define the relationship of permeance with temperature by means of an Arrhenius-type equation:

(5) $$P=P_{0}\cdot e^{\left(\frac{-Ea}{R\;T}\right)}$$

where $P_{0}$ is constant, *Ea* is the activation energy for permeation, *R* is the universal gas constant and *T* is the operation temperature (Kelvin). Figure 13 shows the Arrhenius plot of oxygen and nitrogen permeances versus reciprocal temperature for the Matrimid/PDMS dual-layer hollow fiber membrane when a synthetic mixture of oxygen and nitrogen with a 50:50 mol ratio is fed. For this case, the activation energies for oxygen and nitrogen permeation are 34.9 kJ mol^−1^ and 36.1 kJ mol^−1^, respectively, which explains why the selectivity decreases slightly with increasing temperature. In addition, activation energies have also been obtained for the dual-layer membrane when pure oxygen and nitrogen are fed, obtaining very similar results, 32.9 kJ mol^−1^ for oxygen and 34.8 kJ mol^−1^ for nitrogen. 

### 3.4. Comparison with Previous Studies on Gas Separation Performance

Among the three membranes tested in this work, the Matrimid/PDMS dual-layer membrane is the one that has yielded the best results, and more specifically with working conditions of 80 °C, since the temperature significantly increases the permeance to oxygen and nitrogen without hardly compromising the selectivity (30.8 GPU of O_2_ permeance and 4.7 O_2_/N_2_ selectivity). In order to see how good this performance is, it is necessary to compare it with other studies reported in the literature.

**Figure 14:**
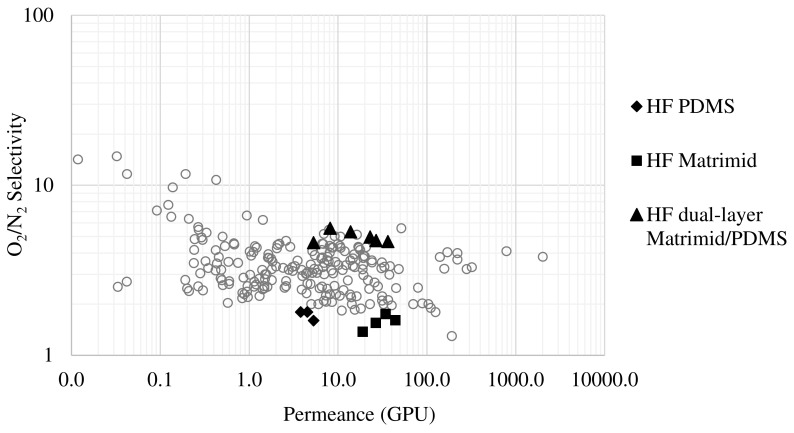
Robeson plot for O2 selective membranes.

In gas permeation, the Robeson plot is commonly used to compare the separation performance of some membranes with others. This is a representation in which the permeance (or permeability) of a membrane towards a gas is plotted against the selectivity it has for that gas. In order to compare the performance of the membranes developed in this work with other studies reported in the literature, Figure 14 shows the Robeson plot, representing the oxygen permeance versus O_2_/N_2_ selectivity, including the literature data available in the Membrane Society of Australasia (MSA) database [50]. This comparison of membrane productivity has been carried out in terms of permeance values (GPU), which is a parameter for which it is not necessary to consider the thickness of the membrane for its calculation. This makes the comparison of the membranes with those reported in the literature more realistic. In this type of graph, the performance of a membrane will be better the higher the permeance and the higher the selectivity, so if we look at the results provided by the dual-layer hollow fiber membrane developed in this work, it can be seen that it is among the best in the literature. Focusing on the possible implementation of these membranes at the industrial level, high permeance is the main goal. Therefore, with the hollow fiber membrane modules developed in this work, the permeance of a material such as Matrimid has been considerably increased in comparison with other works, with a good selectivity. The development of these hollow fiber membranes, which perform well compared to others in the literature, opens up the possibility of their implementation at the industrial level to lower the costs of this separation process, which often combines membrane technology with other technologies such as cryogenic distillation [51,52].

## 4. Conclusions

Oxygen/nitrogen separation constitutes, at present, one of the most interesting separations in the gas separation field. In this work, the performance of various hollow fiber membranes in the separation of oxygen and nitrogen from air was studied in terms of O_2_ permeance (GPU) and O_2_/N_2_ selectivity. Three types of membranes, Matrimid hollow fiber membranes, PDMS hollow fiber membranes (with a PVDF support), and Matrimid-PDMS dual-layer hollow fiber membranes, were developed. The combination of spinning and dip coating methods resulted in mechanically strong and stable hollow fiber membranes with a dense outer layer that allows separation. The novelty of this system is the development of these two methods for the manufacture of membranes in a hollow fiber configuration, which makes it possible to obtain much thinner active layers than with the traditionally used flat-sheet membranes. In addition, with this configuration, it is possible to obtain higher permeance values, which is of vital importance when implementing this technology on an industrial scale. 

It was observed that the Matrimid hollow fiber membrane has very good flux values (31.2 GPU for oxygen at 50 °C) but its O_2_/N_2_ selectivity is compromised (1.6) due to the thin selective dense layer, which is suspected to have micropinholes on its surface. To avoid this effect, a thin layer of PDMS was added to cover these possible defects and also provide a second dense layer for separation. A dense PDMS layer of about 70 µm was studied separately on a porous PVDF support, obtaining O_2_ permeances of 4.5 GPU and O_2_/N_2_ selectivity of 1.8 (at 40 °C). The performance of a Matrimid-PDMS dual-layer hollow fiber membrane was studied, with quite good results of O_2_ permeance (11.7 GPU) and O_2_/N_2_ selectivity (5.3) at 50 °C. It can be concluded that the Matrimid hollow fiber membrane presented small pinholes on its surface and, with the addition of the PDMS layer, it was possible to seal and obtain selectivity values much higher and closer to those of a Matrimid membrane without defects.

Finally, the effect of temperature on the permeance of the dual-layer membrane was studied using an Arrhenius-type equation, obtaining an activation energy of 34.9 kJ/mol for oxygen and 36.1 kJ/mol for nitrogen. Comparing the results obtained with results previously reported in the literature for oxygen/nitrogen separation in a Robeson plot, it can be seen that the Matrimid/PDMS dual-layer hollow fiber membrane is among the best in the literature.

## Figures and Tables

**Table 1:** Parameters of the spinning process.

HF Membrane	PVDF	Matrimid
Dope composition (wt.%)	16	20
Solvent	NMP	NMP
Additive content (wt %)	1	-
Dope flow rate (mL min^−1^)	2	3
Dope extrusion temperature (°C)	20	60
Bore liquid	Ethanol 20 wt.% (Milli-Q water)	NMP 20 wt.% (Milli-Q water)
Bore liquid temperature (°C)	20	20
Bore liquid flow rate (mL min^−1^)	1	3
External coagulant	Water	Water
External coagulant temperature (°C)	30	15
Air gap (cm)	5	6
Take up speed (m min^−1^)	12	12
Coagulation bath depth (m)	1.5	1.5
Inner diameter (μm)	420	580
Wall thickness (µm)	170	50

**Table 2:** Gas permeation experiments conditions.

	Feed Flow (mL/min)	Sweep Gas Flow (mL/min)	Total Feed Pressure (bar)	Total Permeate Pressure (bar)
Matrimid	180–200	5–5.2	1.2–4.5	1
PDMS	180–220	15–30	1.2–4.5	1
Matrimid + PDMS	180–220	5–5.8	1.2–4.5	1

**Table 3:** Permeance and selectivity values of Matrimid hollow fiber membrane.

T (°C)	Pure Gases Feed			Mixture 50:50 Feed		
	P O_2_ (GPU)	P N_2_ (GPU)	α (-) ideal	P O_2_ (GPU)	P N_2_ (GPU)	α (-)
20	-	-	-	15.4	11.8	1.3
30	13.6	8.4	1.6	20.5	14.7	1.4
40	17.7	11.5	1.5	26.9	18.3	1.5
50	22.8	15.3	1.5	31.2	19.8	1.6

**Table 4:** Permeance and selectivity values of PVDF/PDMS hollow fiber membrane (mixture 50:50 feed).

T (°C)	P O_2_ (GPU)	P N_2_ (GPU)	α (-)
30	3.8	2.1	1.8
40	4.5	2.5	1.8
50	5.3	3.4	1.6

**Table 5:** Permeance and selectivity values of Matrimid/PDMS hollow fiber membrane.

T (°C)	Pure Gases Feed			Mixture 50:50 Feed		
	P O_2_ (GPU)	P N_2_ (GPU)	α (-) Ideal	P O_2_ (GPU)	P N_2_ (GPU)	α (-)
30	3.6	0.7	4.8	4.5	1	4.6
40	5.7	1.1	5.3	6.9	1.2	5.6
50	8.4	1.6	5.3	11.7	2.2	5.3
60	11.6	2.6	4.5	19.4	3.9	5
70	-	-	-	22.6	4.8	4.7
80	-	-	-	30.8	6.6	4.7

## Data Availability

The data presented in this study are available from the corresponding author upon reasonable request.
